# A semiparametric Bayesian proportional hazards model for interval censored data with frailty effects

**DOI:** 10.1186/1471-2288-9-9

**Published:** 2009-02-10

**Authors:** Volkmar Henschel, Jutta Engel, Dieter Hölzel, Ulrich Mansmann

**Affiliations:** 1 Institute for Medical Informatics, Biometry and Epidemiology, and Tumour Registry Munich, University of Munich, Marchioninistr, 15, D-81377 Munich, Germany; 2 Institute of Statistics, University of Munich, Ludwigstr, 33, 80539 München, Germany; 3 Biostatistics, Hoffmann-La Roche Basel, PDIB Bau 670/413, Malzgasse 30, CH-4070 Basel, Switzerland

## Abstract

**Background:**

Multivariate analysis of interval censored event data based on classical likelihood methods is notoriously cumbersome. Likelihood inference for models which additionally include random effects are not available at all. Developed algorithms bear problems for practical users like: matrix inversion, slow convergence, no assessment of statistical uncertainty.

**Methods:**

MCMC procedures combined with imputation are used to implement hierarchical models for interval censored data within a Bayesian framework.

**Results:**

Two examples from clinical practice demonstrate the handling of clustered interval censored event times as well as multilayer random effects for inter-institutional quality assessment. The software developed is called survBayes and is freely available at CRAN.

**Conclusion:**

The proposed software supports the solution of complex analyses in many fields of clinical epidemiology as well as health services research.

## Background

Interval-censored survival data occur when the appearance of an event is assessed by means of an examination method that cannot tell the exact time of change in disease status, but only that the change has happened since the last examination. This is in contrast to the standard (naive) thinking that change in status coincides with the time of its first positive examination.

For example, the recurrence of a tumor during the follow-up of a treated cancer patient is an event which happens between two follow-up (FU) examinations. Often it is not possible to connect this event with an exact time (like time of first symptoms, time of first palpable presence, time of death,...) The information for a patient with recurrence is therefore as follows: it is known that up to a certain time (last FU examination at time t1) the patient is free of a recurrence. The recurrence happened between time t1 and t2 (present FU examination at time t2). This is less informative as the usual situation of right censoring. Non- or semi-parametric methods for interval censored data are not frequently used in clinical research papers. The reason may be that these methods are technically more complicated than standard survival methods based on exact or right-censored times. There is a a rich methodological body of methods and algorithms. But there is no easy to use software package in a popular statistical software environment. 

The first frequentist work on interval censored event data is the model of Finkelstein. Finkelstein [1] first introduced a quasi semi-parametric model for regression analysis of interval-censored failure time data based on a finite dimensional maximum likelihood problem. For large datasets the information matrix is sparse and may not be invertible. Therefore, it is often to difficult to derive standard errors of the parameter estimates. Huang [2], Huang and Wellner [3] and Lin, Oakes and Ying [4] present alternative models for semi-parametric regression analysis on interval censored data. Pan [5] proposes the use of the iterative convex minorant algorithm (ICM) to handle the complex likelihood. He avoids the full information matrix and uses a bootstrap procedure to quantify the uncertainty of the inferred regression coefficients. Other approaches to likelihood estimates can be found in the work of Satten [6], or Huber, Solev and Vonta [7]. These methods assume independent observations.

Bellamy et al. [8] extend parametric event time models to clustered and interval censored settings by introducing additive frailties to the linear predictor. Frailty comes into play when multiple events are considered for a given unit. Frailty can also count for unobserved covariates. Bellamy et al. implement their algorithm in existing commercial statistical computing software Bellamy et al. [8]. The authors model dependency between multiple events of a patient by frailty: doctor visits during the subject's time in study. Bellamy's idea is easily implemented in a Bayesian framework. WinBUGS [9] allows to implement analyses for interval censored data with frailty in the same model. A parametric approach via Weibull model or Accelerated Failure Time (AFT) model is easily realized. One can accommodate a frailty to the linear predictor part. But, the implementation of semiparametric proportional hazards models in WinBUGS is cumbersome (see Example Leuk in Example Volume 1 of the WinBUGS software).

Bayesian analysis of event data using non-or semi-parametric models started immediately after Cox [10] with work of Ferguson [11] and Kalbfeisch [12]. A summary of the current state of the art is given in Dey, Müller and Sinha [13] and Ibrahim, Chen and Sinha [14].

Many authors discuss a Bayesian approach to interval censored data with different forms of frailty. They demonstrate the advantageous combination of Bayesian inferential methods and MCMC sampling in this specific setting. The basic strategy is formulated by Härkänen, Virtanen and Arjas [15]. They avoid complex likelihoods by Bayesian data augmentation of censored lifetimes. They study a problem of dentistry where the dependence of event times between the teeth of a subject are accounted for by introducing subject-specific frailty parameters. Given such frailty parameters, the tooth lifetimes are independent. The hazard functions are defined non-parametrically by using piecewise constant functions. Finally, the interval censoring is handled by augmenting the data with unobserved exact event times. The authors also offer software described in Härkänen [16].

Another, more general methodology with similar ingredients has been published by Hennerfeind, Brezger and Fahrmeir [17]. In their work, Hennerfeind et al. do not consider explicitly interval censoring, however this is done by Kneib [18]. Kneib avoids the augmentation approach and handles the likelihood of interval censored observations by numerical integration techniques.

The purpose of the paper is to introduce an algorithm which uses a general software framework for statistical applications http://www.R-project.org and which is easy to apply to a large range of practical applications. We illustrate the theory behind the different modules of the algorithm and describe the specific MCMC strategies used to sample the posterior distribution. We restrict our consideration to specific submodels of Hennerfeind et al. and Kneib which are relevant for specific applications in clinical epidemiology. This is an advantage for many analysts. It bewares of the need to win a full understanding of the more general model classes.

Our approach is structured as follows: The working engine is a Bayesian model for right censored event data which is flexible with respect to the frailty structure underlying the data. We use standard frailty models like log-normal and gamma frailty. Second, we apply a fast data imputation algorithm: a piecewise exponential distributed event times which can easily sampled from piecewise constant hazard functions.

Being Bayesian we have to specify the priori belief in the shape of the baseline hazard which we assume to be smooth. A first order random walk is chosen.

The technical advantage with respect to the data augmentation given by a stepwise baseline hazard function may be offset by its possibly suboptimal fit to the true baseline hazard function. Therefore, we additionally implemented a spline based estimation of the log baseline hazard function. The draw back when performing the data imputation for the interval censored data is the need to linearly approximate the cumulative hazard function.

In Section 'Methods' the methods are described. Subsection 'The basic model' discusses the basic model for right-censored event data. Subsection 'Sampling procedure' describes the sampling procedure needed for estimating the posterior distributions in the basic model. Subsection 'Extensions of the basic model' introduces the technique of data augmentation and the concept of frailty into the model. In Section 'Results' the results are given. Subsection 'Simulation' shows the results of a simulation and Subsection 'Examples' presents two examples, one of current status event data with frailty and one with right censored data and two frailty terms.

## Methods

### The basic model

This subsection sketches a Bayesian approach to a multivariate fixed effects proportional hazards model for right censored data. The specification of its posterior distribution needs the following ingredients: First, the likelihood of the observed data; second, specific prior distributions for regression parameters, hyperparameters, and baseline hazard; third, a Markov Chain Monte Carlo (MCMC) algorithm which can be used to sample the posterior distribution of the parameters of interest.

The data, based on a sample of size *n*, consists of the *n *triples (*t*_*i*_, *δ*_*i*_, **x**_*i*_), *i *= 1,..., *n *where *t*_*i *_is the time on study for subject *i*, *δ*_*i *_is the event indicator for subject *i *(*δ*_*i *_= 1 if the event has occurred, *δ*_*i *_= 0 if the observation is right censored), **x**_*i *_is the *r*-dimensional vector of covariate values for subject *i*.

The basic quantity for likelihood construction is the survival function of an individual surviving beyond time *t*

(1) $$S(t|x)=\exp[-e^{\beta^{\prime }x}\Lambda_{0}(t)]$$

given **x**, the *r*-dimensional covariate vector, and *β*, the *r*-dimensional vector of regression coefficients. Λ_0_(*t*) is the cumulative baseline hazard at time *t*:

(2) $$\Lambda_{0}(t)=\int_{0}^{t}\lambda_{0}(s)\text{d}s.$$

The likelihood contribution of the *i*-th single observation is given by

(3) $$\lambda_{0}{\left(t_{i}|x_{i}\right)}^{\delta_{i}}S(t_{i}|x_{i})=\exp\left\{\delta_{i}[h(t_{i})+\beta^{\prime }x]-{\text{e}}^{\beta^{\prime }x}\int_{0}^{t_{i}}\exp[h(s)]\text{d}s\right\}$$

where *h*(*s*) = ln[*λ*_0_(*s*)] is the log transformed baseline hazard function. The function *h *will be modeled as a stepwise constant function as well as a cubic spline. The stepwise approach results piecewise exponential survival distributions. The use of cubic splines makes the estimated baseline hazard function smoother. Both concepts can be formulated as B-splines, see de Boor [19]. Therefore the time axis [0, ∞) is partitioned into disjoint intervals *I*_*k *_= [*θ*_*k*-1_, *θ*_*k*_) for *k *= 1,..., *K *+ 1.

The times *θ*_*k*_, *k *= 1,..., *K *are chosen to create intervals with comparable information content, i.e. similar number of events. To this end a Kaplan Meier estimator is applied to the right censored data. It is interpolated linearly. Spacing the range of the resulting survival curve into *K *equal parts and using the inverse function of the modified Kaplan Meier estimator for the interval boundaries gives the times *θ*_*k*_, *k *= 1,..., *K*. We observed that a large K destabilized the mixing properties of the MCMC chain by resting in a state for long periods. Therefore, we chose K as large as possible by keeping an acceptable mixing of the chain.

The priors for the components of the vector *β *will be independently normal distributed with mean 0 and a small precision *τ *(Precision is defined as the inverse of the variance: *τ *= $\tau =\frac{1}{\sigma^{2}}$), see Gamerman [20].

The prior for the coefficients of the spline function *h*, the approximated version of the log-transformed baseline hazard function, will be a first order process which gives prior information on smoothness. This is a Bayesian P-spline approach, see Lang and Brezger [21].

The log baseline hazard function *h *is approximated by B-spline functions, $h(t)=\sum_{k=0}^{K}h_{k}b_{j,k}(t)$, where *b*_*j*_,·(*t*) denotes a B-spline function of degree *j*. In our approach *j *is zero for the step functions or three for the cubic splines. The first order process is defined as *h*_*k *_= *h*_*k*-1 _+ *ϵ*_*k *_with *ϵ*_*k *_~ *N*(0, $\sigma_{k}^{2}$) and *h*_0 _~ *N*(0, $\sigma_{0}^{2}$), where *h*_0 _and *ϵ*_*k*_, *k *= 1,..., *K *are pairwise independent. The choice of the mean of the *h*_0 _prior looks quite arbitrary. We try to compensate for this by choosing its variance $\sigma_{0}^{2}$ as quite large. This defines a prior with hardly any influence and allows the data to determine the value of the hazard function at t = 0. The variances for later time points are chosen as $\sigma_{k}^{2}=\Delta_{k}\sigma_{1}^{2}$ and Δ_*k *_may be defined by the mean of the corresponding interval lengths, where the B-splines are different from zero. The inverse of the covariance matrix $\Sigma ={\left(E(h_{k}h_{l}]\right)}_{k,l=0,...,K},\Sigma^{-1}$, can be written as $\frac{1}{\sigma_{0}^{2}}Q_{0}+\frac{1}{\sigma_{1}^{2}}Q_{1}$, where **Q**_0 _is a null matrix except at position (0, 0) where it is 1 and **Q**_1 _is a simple structured band matrix of bandwidth one, due to the first order process.

The parameters $\frac{1}{\sigma_{0}^{2}}$ = *τ*_0 _and $\frac{1}{\sigma_{1}^{2}}$ = *τ*_1 _are treated as hyperparameters with flat gamma priors.

### Sampling procedure

#### Sampling for the parameter vector

Aitkin and Clayton [22] pointed out that the proportional hazards model can be interpreted as a generalized linear model by writing the likelihood function *L *in the form

(4a) $$L=\prod_{i=1}^{n}{\left[f(t_{i})\right]}^{\delta_{i}}{\left[S(t_{i})\right]}^{1-\delta_{i}}$$

(4b) $$=\prod_{i=1}^{n}\left[\mu_{i}^{\delta_{i}}{\text{e}}^{-\mu_{i}}\right]{\left[\frac{\lambda_{0}(t_{i})}{\Lambda_{0}(t_{i})}\right]}^{\delta_{i}}$$

where *μ*_*i *_= Λ_0_(*t*_*i*_) exp(*β*'**x**_*i*_). With a given cumulative baseline hazard Λ_0_(*t*_*i*_) this is the likelihood function of a Poisson sample where the observations are the survival indicators *δ*_*i*_, the link function is the canonical link "log" and there is the offset ln(Λ_0_(*t*_*i*_)).

Gamerman [23] describes how to sample effectively the vector of covariates in generalized linear mixed models in a block updating step. This is a combination of the iterated least squares method (IWLS) with a Metropolis-Hastings sampling.

For the prior a weak informative normal distribution N(0, **R**^-1^), **R **= *σ*^2^**I**_*n *_is chosen. The start of the iteration is with *β *= *β*_0 _and *t *= 1. One has to sample β* from N(**m**^(*t*)^, **C**^(*t*)^) where

(5) **C**^(*t*) ^= [**R**^-1 ^+ **X'W**(*β*^(*t*-1)^)**X**]^-1^,

(6) $$m^{\left(t\right)}=C^{\left(t\right)}X^{\prime }W(\beta^{\left(t-1\right)})\tilde{y}(\beta^{\left(t-1\right)}).$$

β* is accepted with probability *α*(*β*^(*t*-1)^, β*) and then *β*^(*t*) ^= β*, else *β*^(*t*) ^= *β*^(*t*-1) ^and *t *is increased by one.

#### Sampling for the baseline hazard

With the given structure of the log baseline hazard function one has to sample the spline coefficients **h **from a Gaussian Markov Random Field (GMRF). Here we follow Knorr-Held and Rue [24] and Rue [25] and sample the log-baseline hazard in one step. The posterior of **h **is

(7) $$\begin{array}{l} \pi (h|\beta ,\tau_{0},\tau_{1},\text{Data})\propto \exp\{-\frac{1}{2}h^{\prime }\Sigma^{-1}h\\ +\sum_{i=1}^{n}\left[\delta_{i}\sum_{k=0}^{K}h_{k}b_{j,k}(t_{i})-{\text{e}}^{\beta^{\prime }x}\int_{0}^{t_{i}}\exp\left[\sum_{k=0}^{K}h_{k}b_{j,k}(s)\right]\text{d}s\right]\},j\in \{0,3\}. \end{array}$$

Knorr-Held and Rue [24] propose to approximate the exponent by a quadratic form, to use the resulting Gaussian random field (multivariate normal distribution) to sample a proposal for **h**, and to accept or to reject the proposal according to a Metropolis-Hastings step. The cumulative baseline hazard which is part of the exponent can be calculated in a closed form in the case of the degree zero B-splines (step functions). A detailed calculation is given in the appendix [see Additional file 1]. The cumulative baseline hazard in the case of the B-splines of degree three is approximated by the trapezoidal rule because it can not be integrated analytically. The trapezoidal rule results in complex terms which contain the exponent of linear terms of **h**. The calculation of a good quadratic approximation to this terms allows to derive the multivariate normal distribution.

#### Sampling for the dispersion parameters

For the dispersion parameters $\sigma_{0}^{2}$ and $\sigma_{1}^{2}$ a flat gamma prior with rate *κ *and shape *ν *is chosen. The full conditional distribution of $\sigma_{0}^{2}$ is again gamma distributed and has rate $\kappa +\frac{h_{0}^{2}}{2}$ and shape $\nu +\frac{1}{2}$ and the full conditional distribution of $\sigma_{1}^{2}$ has rate $\kappa +\frac{1}{2}h^{\prime }Q_{1}h$ and shape $\nu +\frac{K}{2}$.

### Extensions of the basic model

Data augmentation is used to impute unobserved event times which creates right censored data from interval censored data. An unit specific random effect or frailty term is introduced to the proportional hazards model to account for potential clustering of event times within a statistical unit.

#### Chained Data augmentation

The chained data augmentation algorithm applied to interval censored data imputes candidates of possible event times conditional to the model and the data observed. To obtain the posterior *π*(*ω*|Data), where *ω *= (*β*, **h**, *σ*_0_, *σ*_1_), one proceeds iteratively by generating right censored imputations of event times **T **from the predictive distribution *π*(**T**|*ω*, Data), and calculates *π*(*ω*|**T**, Data), from the augmented data, see Tanner [26].

This suggests that one may draw a value of the parameter vector of interest ω* from *π*(*ω*|T*, Data) where T* is drawn from *π*(**T**|*ω*, Data). In the given case the vector *ω *contains all information on *β*, **h**, *σ*_0 _and *σ*_1_. To initialize the sampling the interval censored event data is treated as right censored (event times in finite intervals are set to the interval midpoint, event time in infinite intervals are considered as right censored) and the basic model (Subsection 'The basic model') is applied. The imputation is applied to all interval censored events. The times of the right censored events are unchanged. The imputation is a straightforward sampling procedure based on the individual survival functions which can be calculated from *ω *for every event in every unit. The individual survival function has to be conditioned on the interval in which the event happens, see also Bebchuk and Betensky [27].

(8) $$P(T<t_{i}|T\in [t_{L_{i}},t_{R_{i}}))=\frac{\exp\{-{\text{e}}^{\beta^{\prime }x_{i}}\Lambda_{0}(t_{L_{i}})\}-\exp\{-{\text{e}}^{\beta^{\prime }x_{i}}\Lambda_{0}(t_{i})\}}{\exp\{-{\text{e}}^{\beta^{\prime }x_{i}}\Lambda_{0}(t_{L_{i}})\}-\exp\{-{\text{e}}^{\beta^{\prime }x_{i}}\Lambda_{0}(t_{R_{i}})\}}$$

The cumulative baseline hazard Λ_0_(*t*) is piecewise linear within the defined intervals for B-splines of degree zero. This implies a piecewise exponential survival distribution and a straight forward sampling of the imputed times. The piecewise linear approximation for B-splines of degree three makes a good proposal for the imputed times. Unfortunately the numerics to calculate an acceptance score relies on an approximation of the numerically not treatable true distribution function. Therefore we skipped the acceptance step.

#### Potential clustering of event times

Unit-specific random effects are used to handle clustering of event times within statistical units. Cluster specific covariates are introduced which takes the value 1 for every observation corresponding to the relevant cluster and 0 else. It is assumed that observation *i *belongs to cluster *j*(*i*). Different frailty distributions can be chosen.

#### Log-normal frailty

Log-normal frailty is difficult to handle in frequentist frailty models, see Hougaard [28]. In the Bayesian setting random effects (frailty terms) are treated like regression coefficients

(9) Λ(*t*_*i*_|**x**_*i*_) = Λ_0_(*t*_*i*_) exp{*β*'**x**_*i *_+ *α*_*j*(*i*)_}.

In case of log-normal frailty these coefficients have a normal prior with mean 0 and variance $\sigma_{\alpha }^{2}$, *α *~ N(0, $\sigma_{\alpha }^{2}$). A non-informative gamma prior is assumed for $\tau_{\alpha }=\frac{1}{\sigma_{\alpha }^{2}}$. For the posterior holds

(10) $$\pi (\alpha |rest)=\exp\left\{\sum_{i=1}^{n}\delta_{i}\alpha_{j(i)}-\sum_{i=1}^{n}\exp\{\alpha_{j(i)}\}\Lambda_{0}(t_{i}){\text{e}}^{\beta^{\prime }x_{i}}-\frac{1}{2\sigma_{\alpha }^{2}}\alpha^{\prime }\alpha \right\}.$$

The sampling follows the ideas of Knorr-Held and Rue [24] as described in Subsection 'Sampling procedure'. The posterior of *τ*_*α *_is again gamma distributed.

#### Gamma frailty

A gamma frailty is proposed by Clayton [29]. The gamma frailty distribution offers technical advantages in the maximum likelihood framework because it allows to express the likelihood as a Laplace transform, see Feller [30]. The cumulative hazard function can be written as

(11) Λ(*t*_*i*_|**x**_*i*_) = *z*_*j*(*i*) _exp{*β*'**x**_*i*_}Λ_0_(*t*_*i*_).

The posterior of **z **is with the gamma prior Ga(*κ*, *p*) again gamma distributed *π*(*z*_*j*_|*rest*) ~ Ga(*ψ*_*j *_+ *κ*, Φ_*j *_+ *p*), where $\Phi_{j}=\sum_{i=1}^{n}\delta_{i}{\text{I}}_{j}(j(i))$ and $\Psi_{j}=\sum_{i=1}^{n}\Lambda_{0}(t_{i}){\text{e}}^{\beta^{\prime }x_{i}}{\text{I}}_{j}(j(i))$. For the gamma prior *κ *= *p *is chosen such that the expected value of the prior is one. The parameter *κ *is updated by a random walk proposal and a Metropolis-Hastings step.

## Results

### Simulation

The purpose of the simulation is to show that the theoretical Bayesian framework gives the expected results given a known true model. Let us summarize shortly the essence of the Bayesian paradigm applied to the hazard function.

We define a prior distribution for the log-baseline hazard as a random walk with mean 0 and a specific covariance structure. Within the Bayesian framework the data changes the prior distribution into the posterior distribution. The Bayesian procedure changes the random walk structure of the log-baseline hazard in a two-fold way:

1.) The constant mean 0 is transformed to a specific form of a drift. The drift of the posterior distribution (which is a random walk) of the log-hazard function inferred by our procedure in the simulation study is shown in the accompanying figure and compares the inferred drift to the true log-baseline hazard function used in the data generation.

2.) The quite variable covariance structure of the prior distribution (large variances) allows that the data have a strong influence on shaping the posterior distribution. Data with a lot of information on the process will remove the variable covariance structure and will create a narrow channel around the true drift where realizations of the random walk can be found which represents the posterior of the log-baseline hazard function. In spite the fact, that the choice of the prior distribution is a random walk with the constant mean 0, the choice of a variable covariance structures allows the estimation procedure to come up with a posterior distribution (again a random walk) whose drift describes sufficient closely the true shape of the log-baseline hazard.

The choice of the mean 0 drift for the prior of the log-baseline hazard is motivated by the idea, that if there is a deviation from a constant hazard the data should produce it in the posterior distribution. Reducing the variance in the covariance structure of the prior distribution has the effect that a deviation of the drift of the posterior distribution from the constant mean 0 drift needs stronger support from the data and a shrinkage effect toward the constant function at 0 is executed.

Of course we could start by giving a drift to prior distribution which is close to the supposed true form of the log-baseline hazard. But we rejected the idea of using informative priors. If the covariance structure of the prior is quite variable then the actual drift of the prior distribution has not a strong influence of the drift calculated for the posterior distribution.

Data from a Weibull model is used to validate the proposed MCMC procedure. At the one hand the Weibull model is parametric at the other hand it fits many practically relevant situations. The hazard function is given by Λ(*t*) = (*t*/*b*)^*a *^with shape *a *and scale *b*. The validation model has four covariates and a gamma frailty. The covariates *X*_1 _and *X*_2 _are binary. *X*_1 _is balanced: 50% of the subjects in our virtual population take value one. The covariate *X*_2 _is unbalanced: 70% of the subjects in our virtual population take value one. The variable *X*_3 _is restricted to a well defined range (uniformly distributed on [-1, 1]). The variable *X*_4 _is standard normal distributed.

Data for a subject is created by independent draws from the relevant distributions (Binomial [1,.5], Binomial [1,.7], Uniform [-1,1], Normal [0,1]). The survival time of a subject is sampled from its individual survival distribution given frailty and covariate vector **x **= (*x*_1_, *x*_2_, *x*_3_, *x*_4_)'.

The parameter vector *β *is chosen to be *β *= (0.5, -0.5, 0.5, 0.5)' which represents effects of relevant size in terms of epidemiology and clinic. The shape of the underlying Weibull distribution is *α *= 0.75 which results in a singularity at zero: a large hazard value at time 0 which decreases over time. The resulting baseline hazard is multiplied by the factor exp{*β*_0_}, *β*_0 _= 0.1.

One thousand observations which are randomly assigned to clusters. The cluster size is random but the number of clusters is fixed: 100, 200, and 500. Each cluster carries a gamma frailty. The frailty is sampled from a gamma distribution with equal rate and shape parameters, Ga(*q*, *q*), with *q *∈ {0.5, 1, 2}. The mean of the gamma distribution is one.

For each subject the interval [0, *t*_*max*_) is randomly divided into five intervals by uniformly sampling five random numbers on [0, *t*_*max*_). If the individual event time is at least *t*_*max *_the event time is taken to be right censored at *t*_*max*_, else the event time is interval censored with the interval it falls in.

In order to make the censoring mechanism independent of the individual survival process *t*_*max *_has to be chosen independent of the individual who has to be censored. Therefore the value of *t*_*max *_is the 0.9-quantile of a specific Weibull distribution with shape *a *= 0.75 (see above) and as scale the median of the individual scales which are derived from the specific covariate configuration and the random effect. The time axis was divided into 50 intervals using the Kaplan Meier procedure as described in Subsection 'The basic model'.

The chain runs through 50 000 cycles. The cubic spline model requires 70+#clusters parameters to be sampled per cycle. The Raftery-Lewis diagnostic, see Mansmann [31], was calculated for regression and baseline hazard coefficients. The Raftery-Lewis diagnostics indicates a burn in of 40 000 cycles to reach convergence for all parameters. 10 000 additional samples of the parameters were taken and thinned by the factor ten. The remaining 1000 samples were used for the analysis.

**Figure 1:**
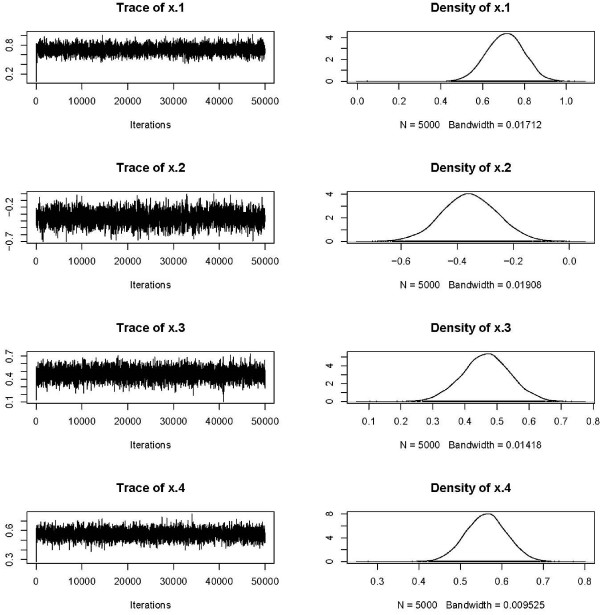
**Traces of the regression coefficients *β*; 100 clusters, rate and shape of the frailty gamma distribution *q *= 1**.

The trace of *β *for one of the scenarios studied can be seen on Figure 1.

The estimates for the regression coefficients *β *and the frailty parameter *q *and their 95% credibility intervals are given in Table 1.

**Table 1:** Simulation: Posterior means and .95 credibility intervals of *β *and *q*

	*q*	0.5			1			2		
#clusters		mean	*q*_0.025_	*q*_0.975_	mean	*q*_0.025_	*q*_0.975_	mean	*q*_0.025_	*q*_0.975_

100	*β*_1_	0.172	-0.030	0.359	0.709	0.534	0.884	0.557	0.396	0.719
	*β*_2_	-0.445	-0.653	-0.234	-0.358	-0.544	-0.165	-0.518	-0.689	-0.346
	*β*_3_	0.573	0.403	0.737	0.466	0.321	0.605	0.566	0.421	0.712
	*β*_4_	0.529	0.418	0.639	0.562	0.465	0.657	0.569	0.479	0.661
	*q*	0.497	0.373	0.645	1.166	0.843	1.595	2.166	1.480	3.120

200	*β*_1_	0.388	0.194	0.590	0.414	0.232	0.591	0.671	0.506	0.849
	*β*_2_	-0.767	-0.984	-0.541	-0.525	-0.730	-0.296	-0.545	-0.733	-0.367
	*β*_3_	0.575	0.385	0.769	0.603	0.440	0.775	0.588	0.439	0.749
	*β*_4_	0.484	0.379	0.595	0.476	0.369	0.574	0.517	0.427	0.610
	*q*	0.554	0.434	0.709	0.953	0.729	1.211	1.821	1.330	2.454

500	*β*_1_	0.484	0.240	0.745	0.615	0.417	0.823	0.594	0.403	0.786
	*β*_2_	-0.588	-0.871	-0.309	-0.519	-0.740	-0.288	-0.359	-0.569	-0.145
	*β*_3_	0.451	0.229	0.660	0.639	0.455	0.817	0.467	0.291	0.645
	*β*_4_	0.473	0.338	0.625	0.621	0.505	0.738	0.500	0.406	0.611
	*q*	0.455	0.362	0.563	0.980	0.739	1.297	1.791	1.288	2.539

The bias for regression coefficients is in general moderate. A pattern for the stronger biased results for the regression coefficients can not be seen in the nine scenarios. The bias for the frailty parameter is in general quite small.

We supposed that increasing the number of clusters (decreasing cluster size) would improve the information content of the data by allowing for more independent observations. Therefore, we expected a clear improvement from 100 clusters to 500 clusters with respect to the precision of estimates. A similar effect was expected from changing the frailty. Reducing the dependency within a cluster should have a similar effect. It was surprising not to see clear trends in the simulation results.

We compared the bias in our strategy to the bias produced by the ICM algorithm proposed by Pan [5], which we provide as R-package intcox. This algorithm is considered as one of the best likelihood based estimation procedures for multivariate interval censored survival data, see Zhang and Jamdhidian [32]. Unfortunately the ICM algorithm is not able to handle frailty. We estimate the parameters by ignoring the clustering but apply clustered bootstrap to calculate their confidence intervals. Table 2 summarizes the estimation of regression coefficients for the different frailty values where the number of clusters is 500. The sample size for the bootstrap confidence intervals was 999.

**Table 2:** Simulation: Estimates and .95 bootstrap confidence intervals of *β *with the intcox procedure

*q*	0.5			1			2		
	estimate	*q*_0.025_	*q*_0.975_	estimate	*q*_0.025_	*q*_0.975_	estimate	*q*_0.025_	*q*_0.975_

*β*_1_	0.363	0.188	0.519	0.360	0.221	0.612	0.415	0.274	0.578
*β*_2_	-0.180	-0.361	-0.037	-0.298	-0.444	-0.066	-0.223	-0.354	-0.053
*β*_3_	0.179	0.045	0.325	0.298	0.175	0.451	0.272	0.159	0.408
*β*_4_	0.186	0.110	0.271	0.362	0.294	0.474	0.319	0.248	0.406

The bias in *β*_1 _is comparable to the MCMC results. The coefficients for the remaining variables show larger bias compared to the MCMC results.

**Figure 2:**
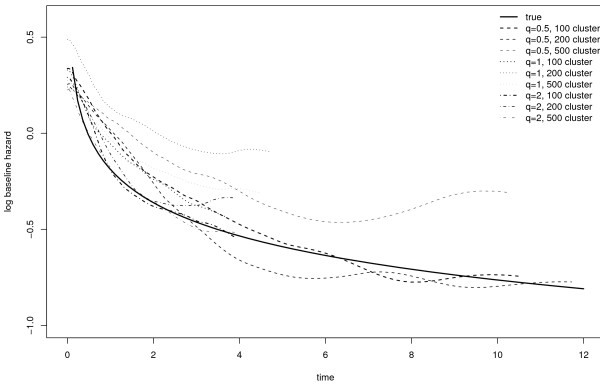
**Estimated and true log baseline hazards**.

The estimates log baseline hazards of the nine onsets are displayed and compared with the true one in Figure 2.

The samples with the larger frailty effect (*q *= 2) give the closest estimates to the true baseline hazard. The Metropolis-Hastings update rates for the coefficients *β*, *h*_*k *_and *q *are about 92, 85 and 97%.

### Examples

In general flat priors are chosen in the R-package survBayes. The precision of the prior for *β*, the rate and shape of the priors for *σ*_0 _and *σ*_1 _and the precision of the random walk for the gamma frailty are set to 0.0001. Possible deviations from these values are indicated below.

#### Aneurisms

Meisel et al. [33] present data on the shrinkage of aneurisms associated with cerebral arteriovenous malformations (cAVM) after embolization treatment. The time to a shrinkage of the aneurism to below 50% of the baseline volume was of interest. Several patients had multiple aneurisms. Each patient was inspected at a random inspection time. Thus, the data is current status censored, the coarsest form of interval censoring (see example 3.4 in Klein and Moeschberger [34]).

Two covariates were considered: the degree of cAMV occlusion by embolization (dichotomized at 50%, variable *mo*) and the location of the aneurism, whether at the midline arteries or at other afferent cerebral arteries, variable *loc*. Multiple aneurisms were observed per patient. In this case the aneurisms share the same "environment" and may not behave independently.

The data set is analyzed with the model described in Section 'Methods'. The log baseline hazard is modeled by cubic splines as well as by a step function. There were phases of no sampling in the Metropolis-Hastings steps for the log baseline hazard coefficients. The problems could be resolved by choosing a not too flat prior with rate and shape q = 0.001 (cubic splines) or q = 0.01 (constant splines) for the smoothness parameter $\sigma_{1}^{2}$. The precision for the random walk for the updating of the gamma frailty parameter *q *was set to 0.01 in the case of the constant splines. 20 000 samples of the parameters were taken and thinned by the factor ten after a burn in of 80 000. The remaining 2000 samples were used for the analysis. There is still no convergence for the last parameters of the log base line hazard while the results for the regression parameters are stable (see Table 3). Inspecting the data shows that only 54 of 149 aneurysms showed shrinkage. This may impair precise estimation of the baseline hazard function for larger times (above 3 years).

**Table 3:** Aneurisms: Summary of posterior distributions and the ICM algorithm for the regression parameters

	cubic			constant			ICM algorithm		
Parameter	P. Mean	*q*_0.025_	*q*_0.975_	P. Mean	*q*_0.025_	*q*_0.975_	Est.	*q*_0.025_	*q*_0.975_

*β*_*mo*_	-1.408	-2.586	-0.451	-1.382	-2.452	-0.451	-1.007	-1.892	-0.417
*β*_*loc*_	-0.879	-1.649	-0.123	-0.868	-1.658	-0.144	-0.831	-1.139	-0.533

**Figure 3:**
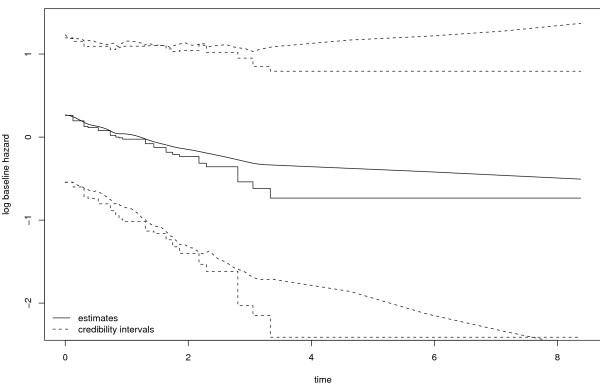
**Log baseline hazard with .95 credibility intervals modeled by cubic and constant splines**.

The estimates and the 95% credibility intervals of the log baseline hazards for both models are shown in Figure 3.

The model with cubic splines gives the smoother fit.

We can compare the Bayesian results with the related maximum likelihood analysis using the Iterated Convex Minorant algorithm as introduced by Pan [5]. The analysis of the Aneurism data set with different likelihood based tools is given in the Vignette accompanying the R-package intcox. The results for the regression parameters are shown in Table 3. The model proposed by Pan [5] considers all observations as independent. This may explain the smaller absolute effect estimates.

#### Colon cancer

The Tumour Register Munich (TRM) collects the data of all cancer patients in Munich and the surrounding southern Bavaria. Here we use data from patients with colorectal carcinoma which includes 9985 patients without metastases collected from 1988 to 2004. Their age distribution at diagnosis is as follows: 621 were less than 50 years old, 1541 between 50 and 60, 2884 between 60 and 70, 2992 between 70 and 80 years old, and 1947 older than 80 years. A total of 5045 patients were male, 4940 female. The distribution of the pT-category 1–4 were 1004, 1482, 5777 and 1374 patients. In 348 patients the pT-category was unknown. 6312 patients had no positive lymph nodes where as in 3224 patients positive nodes were documented. In 449 cases the pN-category was unknown. The grading of the tumour was in 703 cases 1, in 6772 cases 2, in 2117 cases 3 or 4. The grade was unknown in 393 cases. The tumour could removed without a residual in 9631 patients. 1639 patients were treated by a chemotherapy, a neoadjuvant therapy or a combination of chemotherapy and radiation therapy. 8346 patients received no treatment or only a radiation therapy. There were 47 hospitals where the patients were treated. The focus of the analysis is if the institutions influence prognosis. The institutional influence was modeled by a gamma frailty term and not by 47 dummy variables. This avoids arbitrariness related to the aggregation of smaller institution to an artificial unit, Engel et al. [35].

The annual number of patients per clinic in the years 2002 to 2004 were in 34 clinics below 30, in 9 clinics between 30 and 50 and in five clinics more than 50 patients (clinics 2,4,7,25 and 31).

The restricted documentation in clinical tumor registries does not provided all relevant prognostic factors of a patient. That is why a second individual gamma frailty term was introduced. This model can not be fit with standard statistical software.

The data set is analyzed with the model described in Section 'Methods'. The covariates described above are included into the model and hierarchy of frailty terms for clinic and patient is added. The log baseline hazard is modeled by cubic splines. A number of 20 time intervals was chosen. There were sample problems with a flat gamma prior for the smoothness parameter $\sigma_{1}^{2}$. But it is known that the hazard rate of survival is quite constant over time in colon cancer, i.e. there is a small variability of the log baseline hazard. Therefore we chose a informative prior Ga(1,0.1) with a high precision. The remaining parameters were not influenced by the choice of this prior.

After a burn in of 30 000 samples additionally 20 000 samples were taken and thinned by 10. The posterior means and the credibility intervals for the regression parameters are shown in Table 4.

**Table 4:** Summary of posterior distributions for the regression parameters

Parameter	Post. Mean	*q*_0.025_	*q*_0.975_
Age 50–60	0.163	-0.052	0.372
Age 60–70	0.530	0.334	0.736
Age 70–80	1.224	1.023	1.429
Age >= 80	2.081	1.858	2.301
Sex (female)	-0.284	-0.361	-0.204
pT 2	0.086	-0.080	0.266
pT 3	0.493	0.337	0.652
pT 4	1.275	1.088	1.473
pT X	0.185	-0.203	0.539
pN +	0.728	0.630	0.830
pN X	0.823	0.544	1.108
Grade 2	-0.118	-0.275	0.043
Grades 3 and 4	0.058	-0.124	0.241
Grade X	0.003	-0.270	0.287
Residual	0.994	0.804	1.184
Therapy c+	-0.234	-0.356	-0.112

The hazard of death increases with increasing age, pT and pN category and with residual tumour and is lower in women and if a chemotherapy could be applied.

**Figure 4:**
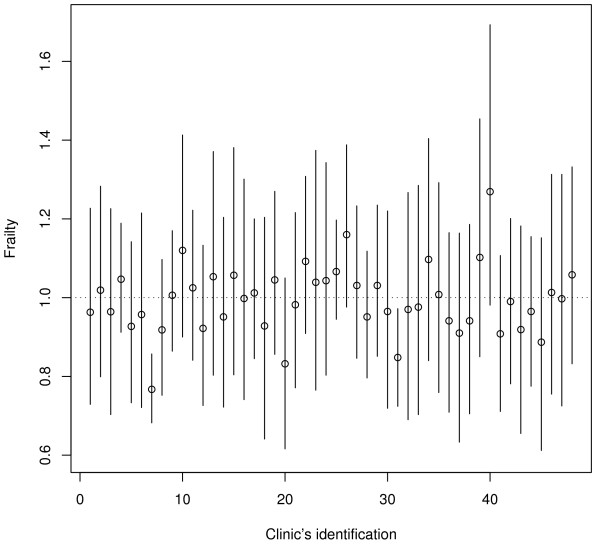
**Posterior means and .95 CI of the frailty coefficients of the clinics**.

The posterior means of the frailty coefficients of the clinics and the 95% credibility interval belonging to them are shown in Figure 4.

The credibility intervals of the clinics with high volume are the smallest. There are only two clinics whose credibility intervals do not include the value one. This value indicates that there is no frailty. Clinics with a high volume tent to have a lower frailty (clinics 7, 31) but this is not true for all of them. On the other hand there is no clear pattern for clinics with lower volume. Clinics with low patient volume may have low frailty (clinics 20, 45) as well as higher frailty (clinics 26, 40).

**Figure 5:**
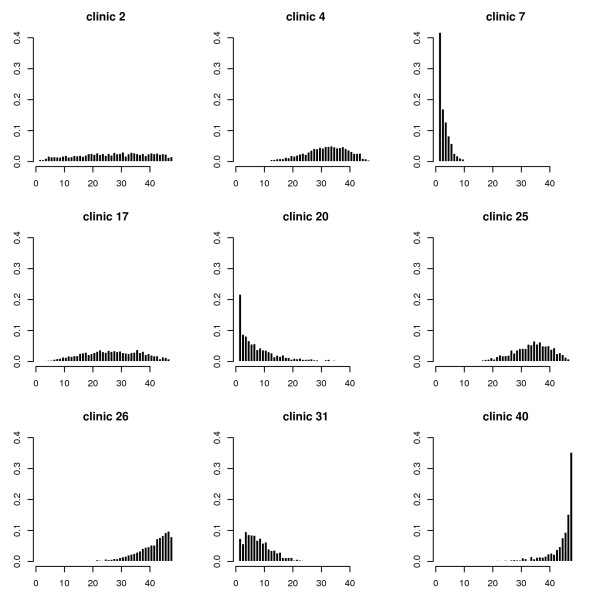
**Sampling of the ranks of selected clinics**.

Transforming the frailty into ranks allows to study the posterior distribution of the rank of a specific clinic. These distributions ar shown for some selected clinics in Figure 5.

The rankings are quite stable for clinics with extreme frailties. Clinics with intermediate frailties range in general over all ranks.

## Discussion

Interval censored event data is a natural documentation scheme for many observational studies. Often, events of interest are not obvious and can only be detected with special diagnostic procedures during follow-up examinations. This is not only of interest for endpoints like disease free survival in oncological studies but also of a broader clinical interest as demonstrated in the aneurysm example. Interval censoring may also help to avoid special forms of bias in epidemiological studies like recall bias – see example 3.4 in Klein and Moeschberger [34]. Including random effects into the model handles unobserved covariates, multiple events, or clustering. Interval censoring and frailty may result in a complex likelihood which is not easy to maximize.

Bellamy et al. [8] showed that within the framework of Weibull models the likelihoods which incorporate interval censoring and simple frailty can be solved by standard software. Formulating the aneurysm example as a Weibull problem in WinBUGS is also possible with a few lines of code:

t.lower[i] <- (1-z[i])*obs.t[i];

t.upper[i] <- [i]*obs.t[i]+(1-z[i])*t.max;

t[i] ~ dweib(r,lambda[i]) I(t.lower[i],t.upper[i]);

log(lambda[i]) <- alpha+b.mo*mo[i]+b.lok*lok[i]+b.ran[gr[i]]

b.ran[gr[i]] ~ dnormal(0, tau)

The treatment is more complex if the statistician decides to use a semi- or non-parametric approach for the hazards model. The example leuk in volume I of the examples which come with the WinBUGS software demonstrates the complexity of a code for a semiparametric Cox-model. Its extension for frailty and interval censoring makes things even more complex. Furthermore, the WinBUGS software is quite restricted in the choice of updating schemes for the MCMC procedure which improve mixing effects of the chains.

Therefore, this paper proposes a Bayesian concept for the simultaneous treatment of frailty and interval censored event times. Data augmentation reduces the interval censored situation to the right censored case and allows to handle frailty in the framework of a semi-parametric proportional hazards model for right censored data. A MCMC procedure was introduced to estimate the parameters of interest. Gamerman's block updating was used to sample the regression coefficients. A block updating based on ideas of Knorr-Held and Rue [24] and Lang and Brezger [21] allowed a simultaneous sample of the baseline hazard function. The incorporation of frailty into the MCMC scheme was straightforward. Data augmentation could make efficient use of piecewise exponential distributions. This is a consequence from a stepwise log baseline hazard function or a piecewise linear approximation to the cumulative hazard function when the log baseline hazard is modeled by cubic splines.

The algorithm is tailored to a wide class of event data problems which are typical for studies in clinical epidemiology, quality control, prognosis, and epidemiological risk assessment. The ideas on which the algorithm is built are also discussed by other groups like Härkänen et al. [15], Kneib [18], Komarek, Lesaffre, Härkänen, Declerck and Virtanen [36], Hennerfeind et al. [17]. The focus of our paper is not to present novel methodology, but to give a full methodological account on a practical algorithm and the accompanying software.

A software which offers comparable functionality is BITE by Härkänen [16]. It is designed for the analysis of event history data using flexible hierarchical models and Bayesian inference. BITE is a stand-alone software while our package uses the full functionality of the R-environment. The handling of BITE is not straight forward. While survBayes is independent of the machine platform, BITE is written for Unix-like operating systems such as Linux. It can also be run on 32-bit Microsoft systems, but a Unix-emulator is needed. Its output files have to be processed with PERL scripts to be read into other statistical software like R or CODA which is needed to present the results of the analysis.

Two examples were worked out. The first example fits in the structure of the model presented in Section 'Methods'. The data consist of multiple current status observations on patients. The example was run with cubic and constant P-splines. The cubic P-spline estimate of the baseline hazard shows comparable results to the constant P-spline in the part of the time spectrum with the most events. The regression parameter estimates also remain comparable. We could compare our results with estimates based on maximum likelihood theory. The Bayesian results are more pronounced compared to the maximum likelihood estimates because the frailty effect is included in the model. This example is in line with the typical problems also discussed by Härkänen [16] and observed in applications of BITE.

The second example presents a two level frailty structure which includes individual frailty of patients but also frailty related to single hospitals. The data describe the survival of colon cancer patients collected in a tumor registry (Engel et al. [35]). The focus of the analysis was on the estimates of the frailty of each individual clinic. A second frailty term is needed to adjust for unobserved individual prognostic covariates. The result indicates that there are remarkable clinic effects in the survival of colon cancer patients. The credibility intervals for the frailties of the clinics with the highest volume are the smallest but for all clinics reasonable estimates of the frailties are obtained. The analysis is of interest for the benchmarking of institutions.

A simulation study based on a Weibull model with three different gamma frailties Ga(*q*, *q*), *q *∈ {0.5, 1, 2}, was used to validate our approach. One thousand events were randomly distributed among 100, 200 or 500 patients (conditioned on the fact that each cluster received at least one event). For the regression parameters we got sensible estimates. The bias for the regression coefficients is in general lower than 20%. No clear pattern for the bias could be detected. A relevant result of the simulation study was the reliable estimation of the frailty parameter which is of primary interest in many problems. The baseline hazard is fitted best for larger frailty values (*q *= 2).

In general we got the impression that the modeling of interval censored event data needs a very careful analysis. Especially convergence of the sampler chains may be not straightforward in small datasets. The proposed procedure needs data sets of sufficient size. Then it will produce in general reasonable estimates of the parameters. But there is always some bias possible with no clear pattern when it happens. Our concept is implemented into the R-package survBayes and is available at the CRAN depository http://www.R-project.org.

## Conclusion

Occurrence of diseases, their progression and death constitute complex event patterns in time. There are two pending problems: (1) There are interval censored events which can only be observed between two examinations. (2) Unspecific and not observable effects have to be taken into account by more or less complex random effect structures. We present a practical solution to both challenges by the software bayesSurv. The paper presents the theory which motivates the algorithms, evaluates the algorithm by a simulation study and applies our approach to two relevant clinical examples. The presented software supports the solution of complex analyses for event data in many fields of clinical epidemiology as well as health services research. It helps to build quantitative models for complex observational data and contributes to an improved quantitative modeling of individual disease histories.

## Competing interests

The authors declare that they have no competing interests.

## Authors' contributions

VH and UM developed the algorithms; VH programmed the software and designed the simulation study; JE and DH provided the data for the colon example and supported its analysis as well as its interpretation, UM proposed and supervised the project, worked on the algorithms, and programmed parts of the software.

## Pre-publication history

The pre-publication history for this paper can be accessed here:

http://www.biomedcentral.com/1471-2288/9/9/prepub

## Supplementary Material

Additional file 1 **Sampling for the baseline hazard (step function approach).** Formal derivation of the sampling for the baseline hazard in the case of degree zero B-splines (step functions). Click here for file
