# Supplementary material for: A semiparametric Bayesian proportional hazards model for interval censored data with frailty effects
Source: BMC Med Res Methodol. 2009 Feb 10;9:9. doi: 10.1186/1471-2288-9-9 (PMC2679769; doi:10.1186/1471-2288-9-9)
Supplement: Additional file 1 — Sampling for the baseline hazard (step function approach). Formal derivation of the sampling for the baseline hazard in the case of degree zero B-splines (step functions). [file 1471-2288-9-9-S1.pdf]

Sampling for the baseline hazard (step function approach)

The integral in the likelihood contribution of the i-th observation can be written as

$$\int_0^{t_i} \exp[h(s)]\mathrm{d}s = \sum_{k=1}^{K} \exp h(\theta_{k-1})[t_i \wedge \theta_k - t_i \wedge \theta_{k_1}] \tag{1}$$

where $t \wedge s = \min\{t, s\}$. The total likelihood of the n observations is given by

$$\begin{aligned} &\prod_{i=1}^{n} \lambda_0(t_i|\mathbf{x}_i)^{\delta_i} S(t_i|\mathbf{x}_i) \qquad (2)\\ &= \exp\left\{\sum_{i=1}^{n}\left\{\delta_i[h(t_i) + \boldsymbol{\beta}'\mathbf{x}] - \mathrm{e}^{\boldsymbol{\beta}'\mathbf{x}} \sum_{k=1}^{K} \exp h(\theta_{k-1})[t \wedge \theta_k - t \wedge \theta_{k_1}]\right\}\right\} \end{aligned}$$

The posterior of $\mathbf{h}$ is

$$\begin{aligned} \pi(\mathbf{h}|\boldsymbol{\beta}, \tau_0, \tau_1, \mathrm{Data}) \propto \exp&\left\{-\frac{1}{2}\mathbf{h}'\boldsymbol{\Sigma}^{-1}\mathbf{h}\right. \qquad (3)\\ + \sum_{i=1}^{n}&\left.\left[\delta_i \sum_{k=1}^{K} h_k \mathrm{I}_{[\theta_{k-1},\theta_k)}(t_i) - \exp\{\boldsymbol{\beta}'\mathbf{x}_i\} \sum_{k=1}^{K} \exp(h_k)[t_i \wedge \theta_k - t_i \wedge \theta_{k-1}]\right]\right\} \end{aligned}$$

This is not a GMRF because of the term $\exp(h_k)$. But this term can be approximated in a neighborhood of h_k as

$$\exp(h'_k) \approx A_i + B_i h'_k + \frac{1}{2} C_i h'^2_k \tag{4}$$

This gives the following GMRF as the proposal density

$$\begin{aligned} q(\mathbf{h}'|\mathbf{h}) &\propto \frac{1}{Z(\mathbf{h})} \exp\left\{-\frac{1}{2}\mathbf{h}'\boldsymbol{\Sigma}^{-1}\mathbf{h} + \sum_{i=1}^{n}\left[\delta_i \sum_{k=1}^{K} h_k \mathrm{I}_{[\theta_{k-1},\theta_k)}(t_i)\right.\right.\\ &\qquad \left.\left. - \exp\{\boldsymbol{\beta}'\mathbf{x}_i\} \sum_{k=1}^{K} (B_i h'_k + \frac{1}{2} C_i h'^2_k)[t_i \wedge \theta_k - t_i \wedge \theta_{k-1}]\right]\right\}\\ &= \exp\left\{-\frac{1}{2}\mathbf{h}'\mathbf{Q}\mathbf{h} + \mathbf{b}'\mathbf{h}\right\} \qquad (5) \end{aligned}$$

with

$$\mathbf{b} = \left(\sum_{i=1}^{n}\left[\delta_i \mathrm{I}_{[\theta_{k-1},\theta_k)}(t_i) - \exp\{\boldsymbol{\beta}'\mathbf{x}_i\} B_i [t_i \wedge \theta_k - t_i \wedge \theta_{k-1}]\right]\right)_{k=1,\ldots,K} \tag{6}$$

$$\mathbf{Q} = \mathrm{diag}\left(-\sum_{i=1}^{n} \exp\{\boldsymbol{\beta}'\mathbf{x}_i\} C_i [t_i \wedge \theta_k - t_i \wedge \theta_{k-1}]\right) - \boldsymbol{\Sigma}^{-1} \tag{7}$$

One can sample easily from (5), see Rue 2001 [25]. One has to compute the band Cholesky decomposition of the precision matrix $\mathbf{Q} = \mathbf{L}\mathbf{L}'$, to solve $\mathbf{L}\mathbf{v} = \mathbf{b}$, $\mathbf{L}'\boldsymbol{\mu} = \mathbf{v}$ and $\mathbf{L}'\mathbf{y} = \mathbf{z}$, where $\mathbf{z}$ is a vector of independent standard Gaussian variables. The sample is the sum $\mathbf{x} = \boldsymbol{\mu} + \mathbf{y}$.

$Z(\mathbf{h})$ is the normalizing constant which depends on $\mathbf{h}$ by the coefficients $\{C_i\}$. The acceptance probability for the Metropolis-Hastings step is $\min(1, R)$, where

$$R = \frac{\pi(\mathbf{h}'|\boldsymbol{\beta}, \tau_0, \tau_1, \text{Data})}{\pi(\mathbf{h}|\boldsymbol{\beta}, \tau_0, \tau_1, \text{Data})} \frac{q(\mathbf{h}|\mathbf{h}')}{q(\mathbf{h}'|\mathbf{h})} \tag{8}$$

To improve the performance of the Metropolis-Hastings step it is recommended not to use the Taylor expansion around h_k in equation (4) but to provide an overall good fit to the full conditional for $\mathbf{h}$ in the region where $\mathbf{h}'$ is expected to be located. Therefore one can use the approximation given by

$$(A_i, B_i, C_i) = \arg\min \left[\int_{h_k-d}^{h_k+d} \left\{ \exp(h'_k) - (A_i + B_i h'_k + \frac{1}{2} C_i h'^2_k) \right\}^2 \mathrm{d}h'_k \right] \tag{9}$$

where d is a crude guess of the step length of h_k to h'_k.
